# Fluorination and chlorination effects on quinoxalineimides as an electron-deficient building block for n-channel organic semiconductors†

**DOI:** 10.1039/c9ra02413a

**Published:** 2019-04-08

**Authors:** Tsukasa Hasegawa, Minoru Ashizawa, Susumu Kawauchi, Hiroyasu Masunaga, Noboru Ohta, Hidetoshi Matsumoto

**Affiliations:** Department of Materials Science and Engineering, Tokyo Institute of Technology 2-12-1 Ookayama, Meguro-ku Tokyo 152-8552 Japan ashizawa.m.aa@m.titech.ac.jp matsumoto.h.ac@m.titech.ac.jp; Department of Chemical Science and Engineering, Tokyo Institute of Technology 2-12-1 Ookayama, Meguro-ku Tokyo 152-8552 Japan; Japan Synchrotron Radiation Research Institute (JASRI)/SPring-8 1-1-1 Kouto, Sayo, Sayo 679-5198 Japan

## Abstract

The quinoxalineimide (QI) unit, containing the electron-withdrawing quinoxaline and imide groups, is an electron-deficient building block for organic semiconductor materials. In this study, three fluorinated or chlorinated QIs (QI-1F, QI-2F, and QI-2Cl), have been designed and developed. We report the impact of the fluorination or chlorination of the QI unit on the electronic structures and charge carrier transport properties as compared to unsubstituted QI (QI-2H) bearing the same *n*-hexyl side chains. The frontier molecular orbital energy levels downshifted with the incorporation of fluorine or chlorine atoms onto the π-framework of QI. Single-crystal structure analyses revealed that all QI-based molecules have an entirely planar backbone and are packed into two-dimensional slipped stacks with diagonal electronic coupling that enables two-dimensional charge carrier transport. Notably, the doubly fluorinated or chlorinated QIs formed compact molecular packing in the single-crystal structures through an infinite intermolecular network relative to unsubstituted QI (QI-2H). The field-effect transistor-based QI molecules exhibited typical n-channel transport properties. As compared to unsubstituted QI (QI-2H), the chlorinated QI exhibited improved electron mobilities up to 7.1 × 10^−3^ cm^2^ V^−1^ s^−1^. The threshold voltages of the fluorinated or chlorinated QI devices were clearly smaller than that of QI-2H, which reflects the lowest unoccupied molecular orbital levels of the molecules. This study demonstrates that the fluorinated or chlorinated QIs are versatile building blocks in creating n-channel organic semiconductor materials.

## Abstract 2

Three fluorinated or chlorinated quinoxalineimide units (QI-1F, QI-2F, and QI-2Cl) have been designed and developed.

## Graphical abstract

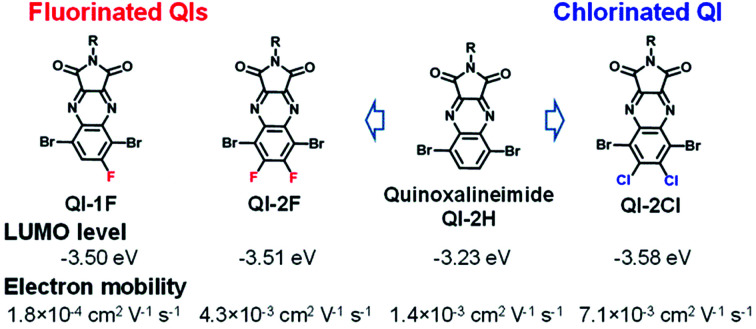

## Introduction

n-Channel organic semiconductors are highly attractive for a wide variety of potential applications including n-channel organic field-effect transistors (OFETs), organic photovoltaics (OPVs), and organic thermoelectrics.^1–5^ However, in contrast to the high performance p-channel materials, n-channel mobilities are still lower than those of p-channel organic materials.^6^ To realize practical organic electronic devices such as complementary-metal-oxide logic circuits, n-channel organic materials with a comparable performance to their p-channel counterparts are essential.^7^ Therefore, tremendous efforts have been devoted to exploring small n-channel molecules and polymers all over the world.

To design an n-channel organic semiconductor, controlling the lowest unoccupied molecular orbital (LUMO) level is a key factor, in which low LUMO levels are desirable for facilitating electron injection from electrodes and enhancing the electrochemical stability of organic materials.^8,9^ The ideal LUMO level for n-channel organic materials ranges from −3.6 to −4.5 eV for effective electron injection from electrodes.^10^ Especially, in order to achieve air-stable electron transport, a LUMO level below −4.0 eV is preferable to prevent electron trapping by oxygen and water.^11–13^

Strong electron-withdrawing groups can pull electrons from the π-conjugated structure. Therefore, the chemical modification with strong electron-withdrawing groups directly contributes to lowering the LUMO levels and effectively stabilizing the reductant species of the π-conjugated molecules. Halogenation, such as fluorination and chlorination, is a simple and effective way to modify the electronic structures and lower the molecular orbital (MO) energy levels because of its strong electron-withdrawing abilities.^14–20^ Especially, the fluorine atom has proven to be highly efficient in improving intramolecular and intermolecular interactions through F⋯H, F⋯F, and F⋯π noncovalent interactions without a considerable steric hindrance.^21–23^ Therefore, fluorination has received much attention in the fields of OFETs and OPVs.^24,25^ As compared to fluorinated π-conjugated materials, a limited number of reports exist on chlorinated materials because of the large size of the chlorine atom, which induces steric hindrance effects in the π-conjugated backbone.^26^ However, chlorine atoms possess attractive properties such as a greater electron density capacity and easier accessibility with a lower cost compared to fluorine atoms.^27^ Even though a chlorine atom possesses less electronegativity than a fluorine atom (Pauling electronegativity for H: 2.20, F: 3.98, and Cl: 3.16),^28^ several studies have reported that chlorination lowers the LUMO level of π-conjugated molecules more effectively than fluorination because of its ability to accommodate a greater electron density.^29–31^

**Scheme 1:**
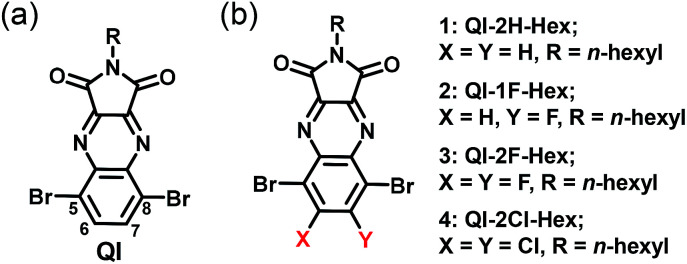
Chemical structures of (a) QI and (b) target QI molecules.

In recent years, we developed the quinoxalineimide (QI-2H) unit containing the electron-withdrawing quinoxaline and imide groups (Scheme 1(a)).^32,33^ The solubility-enabling solution-processed film formation can be controlled by introducing the various side chains in the imide position of the QI backbone. The QI unit possesses two bromides at the 5 and 8-carbon positions of the quinoxaline group, which enable its π-conjugation to expand *via* various chemical modifications such as Stille or Suzuki–Miyaura coupling reactions. Additionally, it is very interesting to systematically investigate the impact of fluorine or chlorine attachments at 6,7-carbons on electronic structure and molecular packing motif of QI framework. In this connection, fluorine and chlorine introductions are quite effective to make novel n-channel building unit in organic semiconducting materials.

In order to examine fluorination or chlorination effects on carrier-transport properties, we now report the synthesis, optical, and electrochemical properties, single-crystal structures, thin-film microstructures, and FET performance of unsubstituted and fluorinated or chlorinated QI molecules 1–4 (QI-2H-Hex (1), QI-1F-Hex (2), QI-2F-Hex (3), and QI-2Cl-Hex (4)) (Scheme 1(b)). The attached hexyl chain ensure solubility in common organic solvents.

## Results and discussion

### Synthesis and characterization

**Scheme 2:**
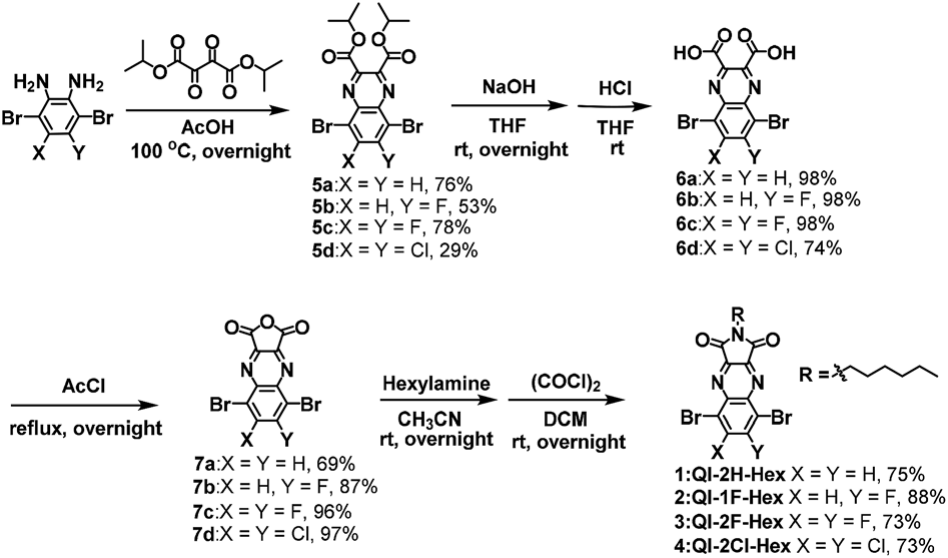
Synthesis of target molecules 1–4.

The synthetic procedure of the target QI-based molecules is illustrated in Scheme 2 and Fig S1.† To prepare the key intermediates 5a–d, diisopropyl 2,3-dioxosuccinate^34^ was condensed with the corresponding dibromobenzene-diamine derivatives.^35–38^ The carboxylic acid compounds 6a–d were obtained by hydrolysis with sodium hydroxide and successive hydrochloric acid treatment. Subsequently, 6a–d were treated with acetyl chloride to provide anhydrides 7a–d. The direct imidization of cyclic acid anhydride with alkylamine in heating *N*,*N*-dimethylformamide or acetic acid is commonly employed to prepare imide part such as naphthalenediimide (NDI).^39,40^ However, in the case of QI, the direct imidization failed; this was likely because of the instability of 7a–d under harsh acidic or basic conditions at high temperatures. Alternatively, the mild reaction condition at room temperature using sequential amidation with the corresponding alkylamine following acid-chloride-mediated cyclization with oxalyl chloride was found to form the target QI-based molecules 1–4 as a pale-yellow solid in relatively high yields (>73%). The detailed synthetic procedures and characterizations are shown in the ESI.† The decomposition temperatures defined by a 5% weight-loss temperature (*T*_5% weight loss_) in thermal gravimetric analysis (TGA) estimated to be 285 °C for 1, 269 °C for 2, 270 °C for 3, and 295 °C for 4 (Fig. S2(a)†). In the differential scanning calorimetry (DSC) profiles under a nitrogen atmosphere (Fig. S2(b)†), 1 only shows an endotherm peak and exotherm peak corresponding to melting and crystallization temperatures of 175 °C and 126 °C, which were determined by the onset peaks, respectively. No marked melting and crystallization behaviors of 2, 3, and 4 would be presumably associated with enhanced fluorine or chlorine bonding of 2, 3, and 4 to make self-assembled structures in the solid state.

### Theoretical calculations

**Fig. 1:**
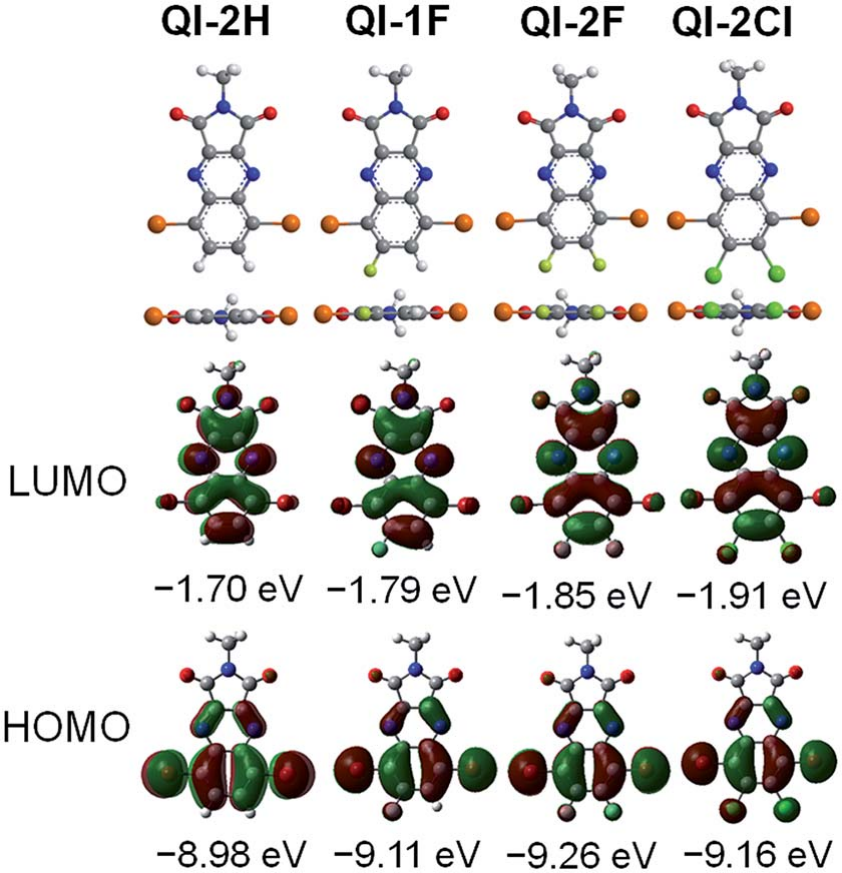
Optimized molecular geometries and the frontier molecular orbital energy levels of methyl-substituted QI-2H, QI-1F, QI-2F, and QI-2Cl estimated from the DFT method at the *ω*B97X-D/6-311+G(d,p).

To investigate the influence of fluorine or chlorine attachments on the optimized geometries and electronic structures, density functional theory (DFT) calculations were performed on the *ω*B97X-D/6-311+G(d,p) level^41^ using the Gaussian 09 program.^42^ To simplify the calculation, the alkyl side chain at the imide position was replaced with the methyl group. Fig. 1 presents the optimized structures and the frontier molecular orbitals. All QI molecules adopt a completely planar structure, and the highest occupied molecular orbitals (HOMOs) are remarkably distributed on the bromine-containing quinoxaline part, while the LUMO orbitals are distributed on the whole QI framework. The estimated HOMO and LUMO levels are −8.98 and −1.70 eV for QI-2H, −9.11 and −1.79 eV for QI-1F, −9.26 and −1.85 eV for QI-2F, and −9.16 and −1.91 eV for QI-2Cl, respectively. Clearly, the fluorine or chlorine substitutions at the 6,7-position of the QI unit could contribute to the reduction of both HOMO and LUMO levels. As compared to the LUMO level of fluorinated QI, that of chlorinated QI is lower despite the larger electronegativity of chlorine. This implies a higher resonance effect of chlorine atoms than fluorine atoms in the QI system.^43^ As a consequence, the electron-withdrawing ability increases in the following order: QI-2Cl > QI-2F > QI-1F > QI-2H. These results reveal that fluorination or chlorination on the QI core is quite effective in tuning the LUMO and HOMO levels without considerable steric effects.

### Optical and electrochemical properties

**Fig. 2:**
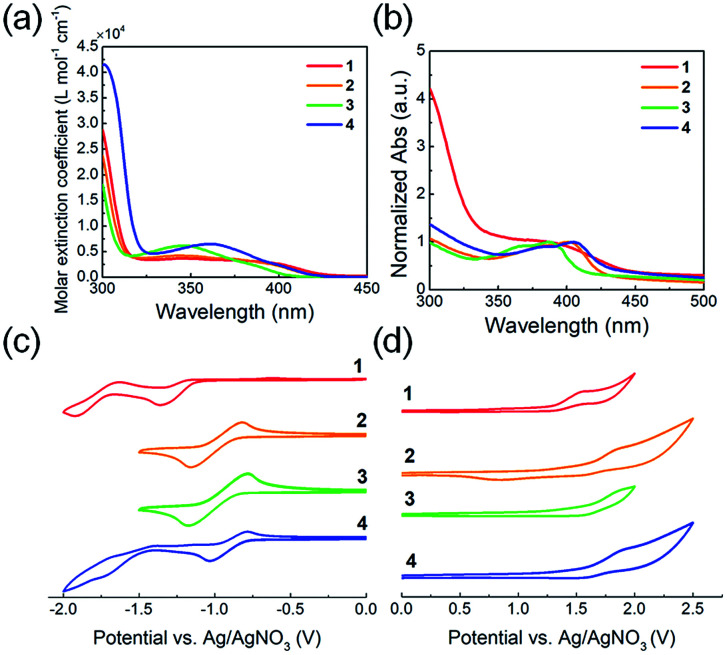
UV-vis absorption spectra of 1–4 for (a) 10^−5^ M chloroform solutions and (b) thin film spin-coated from chloroform solutions. Cyclic voltammograms of molecules 1–4 measured in 0.1 M Bu_4_NPF_6_ in dichloromethane at a scan rate of 100 mV s^−1^: (c) negative scans and (d) positive scans.

The optical and electrochemical properties of the QI-based molecules have been evaluated by UV-vis absorption spectroscopy and cyclic voltammetry (CV). Fig. 2(a and b) shows the UV-vis absorption spectra of chloroform solutions and spin-coated thin films, and the extracted optical data are summarized in Table 1. In the chloroform solutions, molecules 1–4 exhibit broad absorption profiles with peak maxima at around 330–430 nm (Fig. 2(a)). The fluorinated or chlorinated QIs 2–4 exhibit a blue-shifted maximum absorption wavelength (*λ*^sol^_max_) with an increasing maximum molar extinction coefficient (*ε*_max_) compared to that of 1. To further evaluate the electronic transition of QI-based molecules, time-dependent density functional theory (TD-DFT) calculations at the *ω*B97X-D/6-311G+(d,p) were carried out. The absorption data estimated by TD-DFT are listed in Table S1.† From the TD-DFT calculations, the maximum absorption wavelength (*λ*_max_) in the long wavelength region is assigned to the π–π* (HOMO → LUMO) transition. The transition wavelength estimated from TD-DFT (*λ*_transition_) is blue-shifted compared to QI-2H (338 nm) in the following order: QI-2Cl (336 nm) > QI-1F (334 nm) > QI-2F (326 nm), basically agreeing with the experimental observations. The thin-film absorption profiles of molecules 1–4 are red-shifted compared to those of the solutions (Fig. 2(b)). This can be ascribed to the strong π–π intermolecular aggregation in the solid state caused by the highly planar π-framework. The differences between the maximum absorption wavelength (*λ*^film^_max_) and *λ*^sol^_max_ of 2–4 (>19 nm) are slightly larger than that of 1 (11 nm), suggesting that the stronger π–π intermolecular aggregation would be expected in the solid state. The optical energy gaps (*E*^opt^_g_) calculated from the Tauc plot^44^ (Fig. S3†) in the thin film were 2.82 eV for 1, 2.93 eV for 2, 3.03 eV for 3, and 2.89 eV for 4, well agreeing with the trend of the theoretical calculation results.

**Table 1:** Optical and electrochemical properties of molecules 1–4

	*λ* ^sol^_max_ a [nm]	*ε*_max_ × 10^3^^a^ [L mol^−1^ cm^−1^]	*λ* ^film^_max_ b [nm]	*E* ^opt^_g_ c [eV]	*E*_HOMO_ (*E*_onset_)^d^ [eV]	*E*_LUMO_ (*E*_half-wave_)^e^ [eV]	*E* ^CV^_g_ f [eV]
1	376	3.35	387	2.82	−5.80 (1.31)	−3.23 (−1.26)	2.57
2	344	4.21	401	2.93	−6.05 (1.56)	−3.50 (−0.99)	2.55
3	346	6.15	386	3.03	−6.07 (1.58)	−3.51 (−0.98)	2.56
4	361	6.49	405	2.89	−6.04 (1.55)	−3.58 (−0.91)	2.46

a In CHCl_3_ solution.

b Spin-coated thin film.

c Estimated from the Tauc plot.

d Estimated from cyclic voltammetry *vs.* Fc/Fc^+^ (*E*_HOMO_ = −4.80 eV). *E*_onset_ is the onset potential in oxidation curve.

e *E*_half-wave_ is the half-wave potential in reduction curve.

f *E* ^CV^_g_ = *E*_LUMO_ − *E*_HOMO_.

The electrochemical potentials of the LUMO (*E*_LUMO_) and HOMO (*E*_HOMO_) levels were calculated from the onset reduction and oxidation potentials in the cyclic voltammograms (Fig. 2(c and d)), which were calibrated by the ferrocene/ferrocenium (Fc/Fc^+^) couple as a standard work function (−4.8 eV).^45^ The estimated *E*_LUMO_ and *E*_HOMO_ are listed in Table 1. All molecules 1–4 exhibit one or two reversible reduction steps and one irreversible oxidation step. The marked reversible reduction curves indicate the highly stable reduced QI species while the irreversible oxidation curves indicate the relatively unstable oxidized ones. The estimated *E*_LUMO_ and *E*_HOMO_ were −3.23 and −5.80 eV for 1, −3.50 and −6.05 eV for 2, −3.51 and −6.07 eV for 3, and −3.58 and −6.04 eV for 4, respectively. The *E*_LUMO_ values of the fluorinated or chlorinated QIs (2–4) are at least 0.27 eV lower than that of the unsubstituted QI (1), implying that the electron-withdrawing ability lowering their LUMO levels in the following order: 4 > 3 > 2 > 1. Therefore, it is found that the fluorination or chlorination of the QI unit, which induces electron-withdrawing and electronegative properties, provides the QI unit with deep LUMO and HOMO levels, being well consistent with the trend of the theoretical calculations. The HOMO–LUMO energy gaps (*E*^CV^_g_) are estimated to be similar: 2.57 eV for 1, 2.55 eV for 2, 2.56 eV for 3, and 2.46 eV for 4.

### X-ray single-crystal structure analyses

**Fig. 3:**
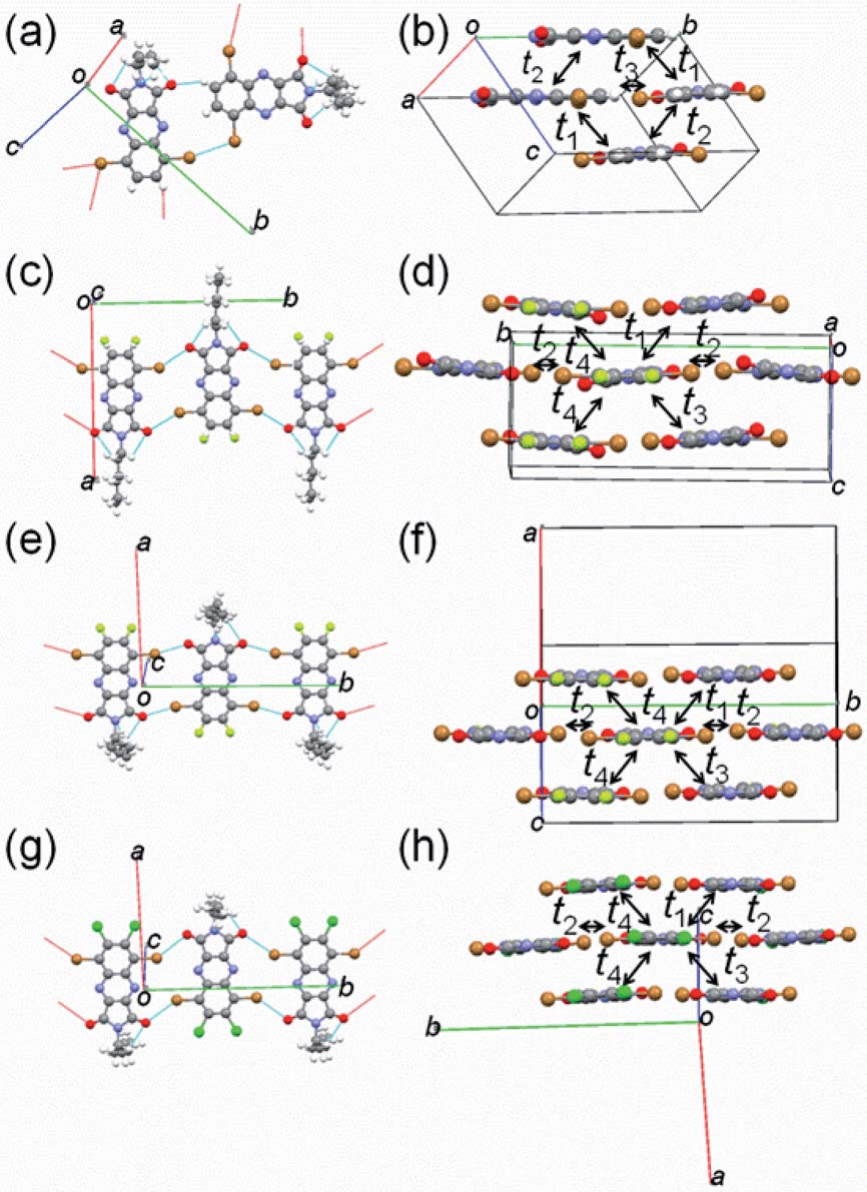
Short contacts with adjacent molecules and molecular packing of (a and b) 1, (c and d) 2, (e and f) 3, and (g and h) 4.

To examine the influence of fluorine or chlorine attachments to the QI core on molecular packing, X-ray single-crystal structure analyses of molecules 1–4 were carried out. The crystallographic data are listed in Table S2,† and the molecular and crystal structures are presented in Fig. 3 and S4–S5.† The obtained single crystals of 1–4 are a large plate-like shape. The fluorine or chlorine containing QIs (2–4) have isostructural structure in the monoclinic system with the space group *P*2_1_/*c*, whereas the unsubstituted QI (1) crystallizes in the monoclinic system with the space group *Pc*. In the structure of 2, the fluorine atom makes a positional disorder at the 6- or 7-position of the benzene ring with occupancies of 44% for F1 and 56% for F2 (Fig. S4(b)†). As expected from the DFT calculations, the π-frameworks of all molecules 1–4 adopt an entirely planar geometry in the structures (Fig. S4†). For all QI molecules, the hexyl chains attached to the imide position extend out of the molecular π-plane, and protons of hexyl group make short intramolecular contacts (≈2.6 Å) between the oxygen and proton (O⋯H) (Fig. 3(a–d)). Molecule 1 forms the short intermolecular Br⋯Br (3.5 Å) and O⋯H (2.4 Å) contacts (Fig. 3(a)). Note is that unsubstituted free protons at the 6- and 7-carbon positions of benzene ring contribute to making H-bonding, leading to form zigzag molecular array along the *a* axis. Meanwhile, molecules 2–4 form infinite short intermolecular contacts through oxygen and bromide (O⋯Br) (2.9–3.2 Å) along the *b*-axis (Fig. 3(b–d)), in which molecules are arranged in the upside-down manner. Interestingly, there are no obvious short contacts utilizing fluorine or chlorine atoms. The estimated interplanar spacings of molecules 2–4 are in the range of 3.1–3.8 Å, which are remarkably shorter than that of molecule 1 (4.46 Å) (Fig. S5†). These observations indicate that fluorine or chlorine attachments on the QI core appears to be useful for forming compact molecular packing through the infinite intermolecular network relative to molecule 1. All molecules 1–4 are packed into two-dimensional slipped stacks along the *c*-axis with diagonal electronic couplings that enable two-dimensional charge carrier transport (Fig. 3(b)). Assuming the tight binding method,^46^ the calculated LUMO and HOMO transfer integrals (*t*_1_–*t*_4_) along the stacks are listed in Table 2. In all molecules, the transfer integrals of the LUMO levels are clearly larger than those of the HOMO levels, suggesting that electron transport is dominant. The calculated transfer integrals are indicative of a two-dimensional carrier transport path.

**Table 2:** Calculated LUMO and HOMO transfer integrals of 1–4^a^

		*t*_1_	*t*_2_	*t*_3_	*t*_4_
1	LUMO	27	6	13	N.A.
	HOMO	1	1	3	N.A.
2	LUMO	34	4	24	32
	HOMO	14	0	17	1
3	LUMO	12	1	12	11
	HOMO	2	3	2	2
4	LUMO	28	8	39	17
	HOMO	15	1	3	4

a Estimated from tight binding method.

### Morphology and microstructure of thin films

**Fig. 4:**
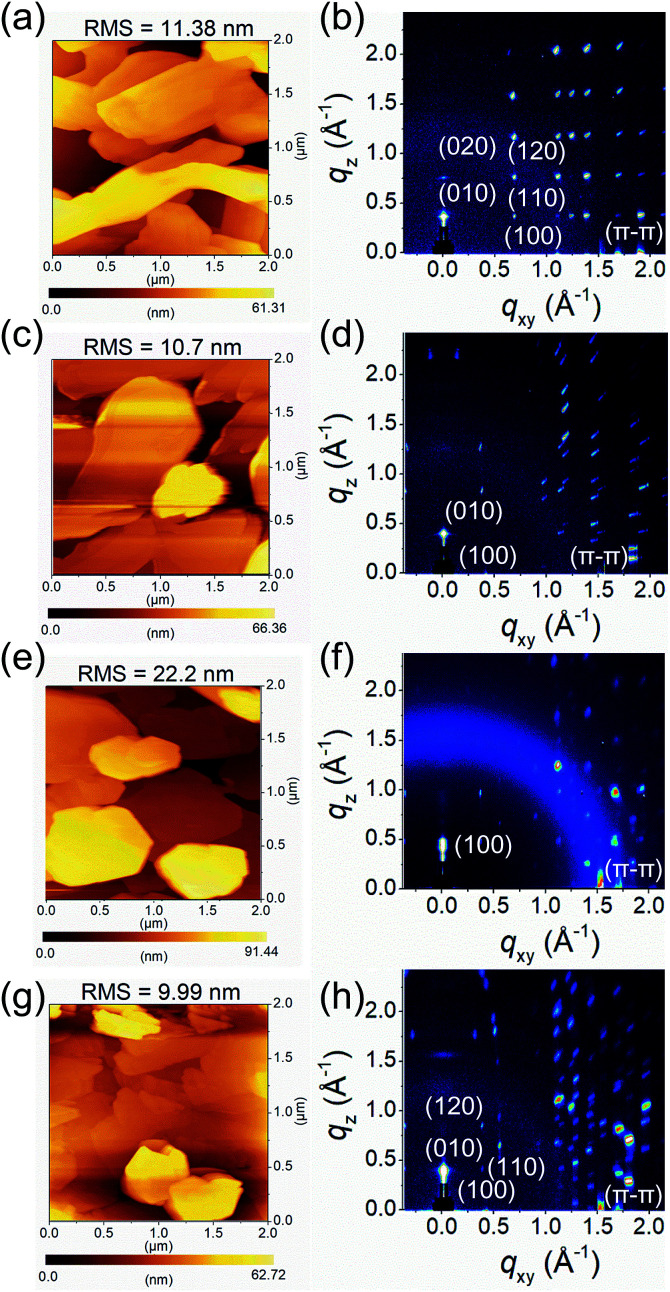
AFM images and GIWAXS patterns of (a and b) 1, (c and d) 2, (e and f) 3, and (g and h) 4 thin films thermally evaporated on TTC (20 nm)-modified substrates, respectively.

To investigate the fluorinated or chlorinated effects of QI molecules on the thin-film surface morphology and microstructures, tapping-mode atomic force microscopy (AFM) and grazing incidence wide-angle X-ray scattering (GIWAXS) were measured. The thin films (45 nm) of molecules 1–4 were thermally deposited on tetratetracontane (C_44_H_90_, TTC)-modified Si/SiO_2_ substrates. The obtained AFM images and GIWAXS patterns are shown in Fig. 4 and S6,† and the corresponding data are summarized in Table S3.† In the AFM images, the surface morphologies of the thin films of molecules 1–4 consist of disconnected large plate-like grains, with high root-mean-square (RMS) values of about 10–20 nm (Fig. 4(a, c, e, and g)). These poor surface morphologies are unfavorable for carrier transport. Interestingly, there is no influence on the grain shape or surface morphology with the introduction of the fluorine and chlorine atoms, implying that the QI core itself have strong tendency to form crystalline thin films.

From the GIWAXS patterns, all molecules show intense and multiple scattering peaks, indicating the high crystallinity of the thin films (Fig. 4(b, d, f, and h)). The corresponding *d*-spacing of the primary peak along the out-of-plane direction is 16.6 Å (*q*_*z*_ = 0.38 Å^−1^) for 1, 15.3 Å (*q*_*z*_ = 0.41 Å^−1^) for 2, 13.6 Å (*q*_*z*_ = 0.46 Å^−1^) for 3, and 16.1 Å (*q*_*z*_ = 0.39 Å^−1^) for 4. Compared to the crystal lattice estimated from single-crystal X-ray analyses, the *d*-spacing values are close length of the *b*-axis for 1 (16.2 Å), 2 (16.4 Å), and 4 (16.3 Å), and the *a*-axis for 3 (13.5 Å). Moreover, in the in-plane direction, all molecules display multiple π–π stacking peaks at *q*_*xy*_, and the corresponding *d*-spacing of 3.73 Å (*q*_*xy*_ = 1.69 Å^−1^) for 1, 3.60 Å (*q*_*xy*_ = 1.74 Å^−1^) for 2, 3.60 Å (*q*_*xy*_ = 1.74 Å^−1^) for 3, 3.69 Å (*q*_*xy*_ = 1.70 Å^−1^) for 4, respectively. In the *q*_*xy*_ direction, molecules 1, 2, and 4 displayed intense peaks corresponding to the *a*-axis with the *d*-spacings are 5.60 Å for 1, 14.8 Å for 2, and 14.6 Å for 4. Although it is difficult to identify the exact molecular orientation, when considering the molecular lengths estimated from X-ray single-crystal structure analyses, these observations indicate that the crystallographic *ac* planes are aligned parallel to the substrate for 1, 2, and 4, whereas the crystallographic *bc* plane for 3 is parallel to the substrate. In the consideration of the results of GIWAXS measurement, the molecular short axes of 2 and 4, which make side-by-side intermolecular infinite network through Br⋯O short contact, are almost perpendicularly aligned on the substrate, while the molecular long axis of 3 is largely tilted on the substrate. However, the molecular long axis of 1, which forms zigzag array along the crystallographic *a*-axis, is normal to the substrate with subtle intermolecular overlap. The resultant molecular tilt angles perpendicular to the substrate are *ca.* 3° for 1, 3° for 2, 45° for 3, and 2° for 4 (Fig. S7†).

### OFET performance

**Fig. 5:**
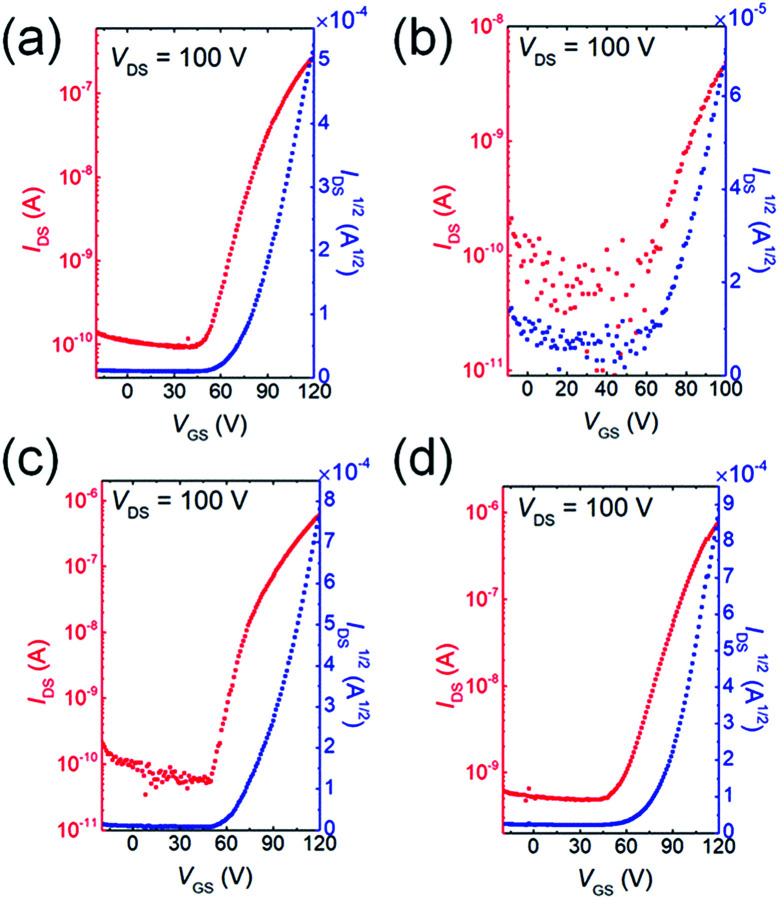
The n-channel transfer curves of (a) 1, (b) 2, (c) 3, and (d) 4, respectively.

To investigate how the fluorine or chlorine of the QI units influence the carrier transport properties, bottom-gate/top-contact OFET devices were fabricated. The thin films of QIs were thermally deposited on TTC-modified Si/SiO_2_ substrates (TTC was used as passivation layer). A highly ordered TTC layer can enhance the crystallinity of the organic semiconductor layer, and thus improve the electron transport property.^47–49^Fig. 5 and S8† show the transfer and output curves, and the corresponding OFET data are summarized in Table S4.† The OFETs based on all molecules show typical n-channel FET characteristics under vacuum conditions. The average electron mobilities (*μ*_e,avg_) and *V*_th_ are 1.4 × 10^−3^ cm^2^ V^−1^ s^−1^ and 77.4 V for 1, 1.8 × 10^−4^ cm^2^ V^−1^ s^−1^ and 69.4 V for 2, 4.3 × 10^−3^ cm^2^ V^−1^ s^−1^ and 69.4 V for 3, and 7.1 × 10^−3^ cm^2^ V^−1^ s^−1^ and 67.6 V for 4, respectively. Fluorination and chlorination at both 6- and 7-positions on QI core (3 and 4) slightly improve their mobilities compared with unsubstituted QI (1). However, singly fluorine-substituted QI (2) displayed lower *μ*_e,avg_, which is owing to the positional fluorine disorder in the solid state.^50^ The *V*_th_ values of the devices of 2, 3, and 4 were clearly smaller than that of the device of 1, which is basically consistent with the decreased the LUMO levels. Therefore, deeper LUMO levels enable the effective electron injection from the gold electrodes, and achieve the low *V*_th_ beneficial for low voltage operation. However, these electron mobilities are much lower than those of the conventional n-type organic semiconductors because of small intermolecular overlap coming from their short π-frameworks. The π-framework extension by means of various coupling reactions utilizing bromines at 5- and 8- positions would further improve electron-transport. Therefore, fluorine or chlorine containing QIs are a useful electron-deficient building block constituting oligomer and polymer semiconductors.

## Conclusions

In this study, we have synthesized QI-based molecules and explored their structure–property correlations. The thermal properties improve by fluorination or chlorination because of the fluorine or chlorine bonding in the solid state. Theoretical calculations suggest that all QI-based molecules have a completely planar framework, and the LUMO levels decrease in the order of QI-2H > QI-1F > QI-2F > QI-2Cl. As compared to unsubstituted QI (1), the absorption profiles of fluorinated or chlorinated QIs (2–4) blue-shift owing to the weakened π-conjugation in the π-framework caused by the strong electron-withdrawing abilities of fluorine and chlorine atoms. The electrochemical measurements reveal that the frontier molecular orbital energy levels downshift with the incorporation of fluorine or chlorine atoms onto the π-framework of QI. In the single crystals, fluorination or chlorination on the QI core appears to be useful for forming compact molecular packing through the infinite intermolecular network relative to unsubstituted QI. The OFET based on all molecules exhibits n-channel transport, and the threshold voltages change with their LUMO levels. Among QI molecules, chlorinated QI (4), with the deepest LUMO level, shows the highest n-channel mobility up to 7.8 × 10^−3^ cm^2^ V^−1^ s^−1^ under vacuum conditions.

We emphasize that exploring versatile electron-deficient building blocks in organic synthesis is very significant for obtaining n-channel high-performance semiconducting oligomers and polymers.

## Conflicts of interest

There are no conflicts to declare.

## Supplementary Material

RA-009-C9RA02413A-s001

RA-009-C9RA02413A-s002
