# Supplementary material for: Fluorination and chlorination effects on quinoxalineimides as an electron-deficient building block for n-channel organic semiconductors
Source: RSC Adv. 2019 Apr 8;9(19):10807–13. doi: 10.1039/c9ra02413a (PMC9062530; doi:10.1039/c9ra02413a)
Supplement: RA-009-C9RA02413A-s001 [file RA-009-C9RA02413A-s001.pdf]

Electronic Supplementary Material (ESI) for RSC Advances.
This journal is © The Royal Society of Chemistry 2019

Electronic Supplementary Information

Fluorination and Chlorination Effects on Quinoxalineimides as an Electron-Deficient Building Block for n-Channel Organic Semiconductors

Tsukasa Hasegawa,[a] Minoru Ashizawa,*[a] Susumu Kawauchi,[b] Hiroyasu Masunaga,[c] Noboru Ohta,[c] Hidetoshi Matsumoto*[a]

[a] Department of Materials Science and Engineering, Tokyo Institute of Technology, 2-12-1 Ookayama, Meguro-ku, Tokyo 152-8552, Japan

[b] Department of Chemical Science and Engineering, Tokyo Institute of Technology, 2-12-1 Ookayama, Meguro-ku, Tokyo 152-8552, Japan

[c] Japan Synchrotron Radiation Research Institute (JASRI)/SPring-8, 1-1-1 Kouto, Sayo, Sayo 679-5198, Japan

Corresponding Authors

*ashizawa.m.aa@m.titech.ac.jp

*matsumoto.h.ac@m.titech.ac.jp

Contents of Supporting Information

I. General ········· S-2

II. Material Synthesis ········· S-3

III. Thermal Properties ········· S-11

IV. DFT Calculations ········· S-12

V. Optical Properties ········· S-13

VI. X-ray single-crystal structure analyses ········· S-14

VII. Fabrication and Characterization of Organic Field-Effect Transistors ········· S-18

References ········· S-23

I. General

All the chemicals and solvents were of reagent grade, were purchased from Tokyo Kasei Chemical Industries, FUJIFILM Wako Pure Chemical Corporation, and Sigma-Aldrich and were used without further purification. All reactions were carried out under an argon atmosphere. The ^{1}H and ^{13}C NMR spectra were recorded using a JEOL JNM-AL300 spectrometer (300 MHz) in deuterated chloroform at 298 K, and tetramethylsilane was used as the internal standard. Cyclic voltammetry (CV) was carried out using an ALS/CH Instruments Electrochemical Analyzer Model 700C. The measurement was performed in dehydrated dichloromethane solution containing 0.1 M n-Bu_4NPF_6 as the supporting electrolyte using a glassy carbon working electrode, a platinum counter electrode, and an $Ag/AgNO_3$ electrode as a reference electrode at a scan rate of 100 mV s^{-1}. From the onset oxidation ($E_{ox,\ onset}$) and reduction ($E_{red,\ onset}$) potentials, the HOMO and LUMO levels were estimated by assuming the ferrocene/ferrocenium energy level to be -4.8 eV under vacuum conditions. The elemental analyses were performed at the Center for Advanced Materials Analysis, Tokyo Institute of Technology.

II. Material Synthesis

3,6-dibromobenzene-1,2-diamine[1], 3,6-dibromo-4-fluorobenzene-1,2-diamine[2], 3,6-dibromo-4,5-difluorobenzene-1,2-diamine[3], 3,6-dibromo-4,5-dichlorobenzene-1,2-diamine[4], diisopropyl 2,3-dioxosuccinate[5], diisopropyl 5,8-dibromoquinoxaline-2,3-dicarboxylate (**5a**)[6], 5,8-dibromoquinoxaline-2,3-dicarboxylic acid (**6a**)[6], and 5,8-dibromofuro[3,4-*b*]quinoxaline-1,3-dione (**7a**)[6] were prepared by following the reported procedures. All reactions were carried out under an argon atmosphere.

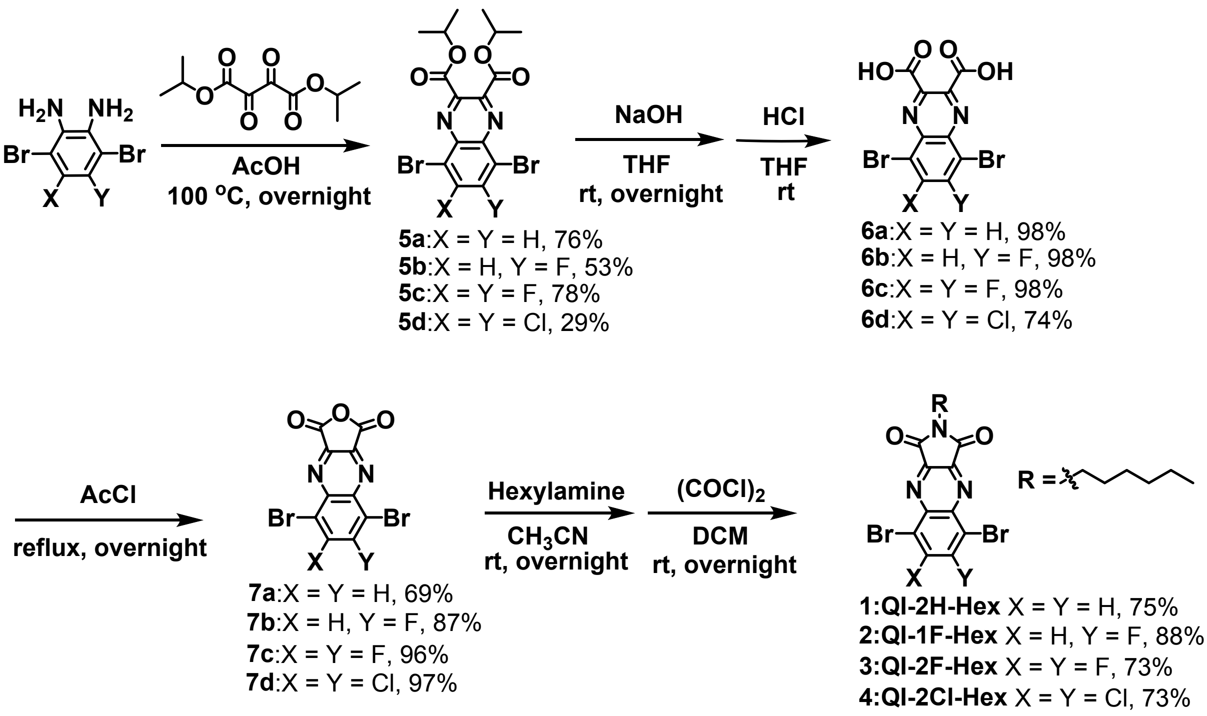

Figure S1 Synthetic route of molecules **1**-**4**.

Synthesis of diisopropyl 5,8-dibromo-6-fluoroquinoxaline-2,3-dicarboxylate (5b)

3,6-dibromo-4-fluorobenzene-1,2-diamine (1.85 g, 6.52 mmol) and diisopropyl 2,3-dioxosuccinate (3.60 g, 15.6 mmol) were dissolved in acetic acid (AcOH) (30 mL) under an argon atmosphere. The resulting

mixture was heated to 100 ^{o}C and stirred for overnight. After cooling to room temperature, the reaction mixture was poured into 100 mL distilled water and extracted with chloroform three times. The organic layer was washed with distilled water twice and then dried with magnesium sulfate ($MgSO_4$). After the removal of the solvent under reduced pressure, the residue was purified by silica gel column chromatography, eluting with dichloromethane (DCM) : hexane (3: 2) to provide 1.64 g (53%) as a yellow solid. ^{1}H NMR (300 MHz, $CDCl_3$, δ, ppm, 25 °C): 8.06 (d, J = 8.1 Hz, 1H), 5.43-5.34 (m, 2H), 1.45 (d, J = 6.3 Hz, 12H). ^{13}C NMR (75 MHz, $CDCl_3$, δ, ppm, 25 °C): 163.55, 163.38, 162.56, 159.15, 146.39, 144.57, 144.52, 140.75, 140.67, 137.25, 137.23, 126.06, 125.68, 125.34, 125.19, 109.08, 108.80, 71.38, 71.33, 21.78. MS(EI) *m/z*: 478 $[M]^+$ Elemental anal. calcd. for $C_{16}H_{15}Br_2FN_2O_4$: C, 40.19; H, 3.16; Br, 33.42; F, 3.97; N, 5.86; O, 13.39. Found: C, 40.02; H, 3.21; Br, 33.09; F, 3.71; N, 5.81.

Synthesis of diisopropyl 5,8-dibromo-6,7-difluoroquinoxaline-2,3-dicarboxylate (5c)

Compound **5c** was synthesized from 3,6-dibromo-4,5-difluorobenzene-1,2-diamine (0.88 g, 2.91 mmol) using the same procedure for **5b** and purified by silica gel column chromatography, eluting with dichloromethane (DCM) : hexane (4: 1) to provide 1.13 g (78%) as a yellow solid. ^{1}H NMR (300 MHz, $CDCl_3$, δ, ppm, 25 °C): 5.43-5.35 (m, 2H), 1.46 (d, J = 6.0Hz, 12H). ^{13}C NMR (75 MHz, $CDCl_3$, δ, ppm, 25 °C): 163.36, 154.09, 153.83, 150.62, 150.25, 145.57, 137.35, 137.31, 110.57, 110.46, 110.42, 110.30, 71.46, 21.77. MS(EI) *m/z*: 496 $[M]^+$ Elemental anal. calcd. for $C_{16}H_{14}Br_2F_2N_2O_4$: C, 38.74; H, 2.84; Br, 32.21; F, 7.66; N, 5.65; O, 12.90. Found: C, 38.43; H, 2.91; F, 7.87; N, 5.55.

Synthesis of diisopropyl 5,8-dibromo-6,7-dichloroquinoxaline-2,3-dicarboxylate (5d)

Compound **5d** was synthesized from 3,6-dibromo-4,5-dichlorobenzene-1,2-diamine (1.07 g, 3.19 mmol) using the same procedure for **5b** and purified by silica gel column chromatography, eluting with dichloromethane (DCM) : hexane (4: 1) to provide 0.49 g (29%) as a yellow solid. ^{1}H NMR (300 MHz, $CDCl_3$, δ, ppm, 25 °C): 5.44-5.36 (m, 2H), 1.46 (d, J = 6.0 Hz, 12H). ^{13}C NMR (75 MHz, $CDCl_3$, δ, ppm, 25 °C): 163.32, 145.97, 138.98, 138.92, 125.93, 71.53, 21.81. MS(EI) *m/z*: 529[M]$^{+}$ Elemental anal. calcd. for $C_{16}H_{14}Br_2Cl_2N_2O_4$: C, 36.33; H, 2.67; Br, 30.21; Cl, 13.40; N, 5.30; O, 12.10. Found: C, 36.26; H, 2.75; Cl, 13.62; N, 5.26; O, 12.08.

Synthesis of 5,8-dibromo-6-fluoroquinoxaline-2,3-dicarboxylic acid (6b)

Compound **5b** (0.60 g, 1.25 mmol) was dissolved in THF (10 mL) under an argon atmosphere. 2 M NaOH aqueous (3.75 mL, 7.50 mmol) was slowly added to the resulting solution and stirred for overnight. After the reaction, the reaction mixture was acidized with 1 M HCl aqueous (8.00 mL, 8.00 mmol). The reaction mixture was poured into 100 mL distilled water and extracted with diethyl ether three times. The organic layer was washed with distilled water twice and dried with $MgSO_4$. After the removal of the solvent under reduced pressure, the residue solid was washed with hexane to provide 0.48 g (ca. 98%) as a yellow solid. The crude product was suitable for use in the next step. ^{1}H NMR (300 MHz, acetone, δ, ppm, 25 °C): 11.2 (br, 2H), 8.44 (d, J = 8.4 Hz 1H). ^{13}C NMR (75 MHz, acetone, δ, ppm, 25 °C): 164.77,

164.60, 162.65, 159.28, 146.57, 144.67, 144.62, 140.77, 140.69, 137.41, 126.35, 125.95, 125.14, 124.98, 108.59, 108.30. MS(EI) *m/z*: 394 $[M]^+$

Synthesis of 5,8-dibromo-6,7-difluoroquinoxaline-2,3-dicarboxylic acid (6c)

Compound **6c** was synthesized from compound **5c** (0.61 g, 1.23 mmol) using the same procedure for **6b**. and washed with hexane to provide 0.50 g (ca. 98%) as a yellow solid. ^{1}H NMR (300 MHz, acetone, δ, ppm, 25 °C): 9.56 (br, 2H). ^{13}C NMR (75 MHz, acetone, δ, ppm, 25 °C): 164.65, 154.17, 153.91, 150.74, 150.48, 145.75, 137.62, 110.27, 110.12, 110.00. MS(EI) *m/z*: 412 $[M]^+$

Synthesis of 5,8-dibromo-6,7-dichloroquinoxaline-2,3-dicarboxylic acid (6d)

Compound **6d** was synthesized from compound **5d** (0.45 g, 0.85 mmol) using the same procedure for **6b** and washed with hexane to provide 0.28 g (ca. 74%) as a yellow solid. ^{1}H NMR (300 MHz, acetone, δ, ppm, 25 °C): 6.43 (br, 2H). ^{13}C NMR (75 MHz, acetone, δ, ppm, 25 °C): 165.21, 146.96, 139.98, 138.65, 126.51. MS(EI) *m/z*: 445 $[M]^+$

Synthesis of 5,8-dibromo-6-fluorofuro[3,4-b]quinoxaline-1,3-dione (7b)

Compound **6b** (0.48 g, 1.22 mmol) and acetyl chloride (10 mL) were refluxed for overnight under an argon atmosphere. After cooling to room temperature, the solvent was removed under reduced pressure and washed with hexane to provide 0.41 g (ca. 87%) as a yellow solid. The crude product was suitable for use

in the next step. ^{1}H NMR (300 MHz, acetone, δ, ppm, 25 °C): 8.70 (d, J = 8.4 Hz 1H). ^{13}C NMR (75 MHz, acetone, δ, ppm, 25 °C): 163.98, 160.56, 158.90, 158.86, 146.25, 145.03, 144.98, 144.57, 144.49, 141.18, 128.75, 128.34, 126.75, 126.59, 110.36, 110.07. MS(EI) *m*/*z*: 376 $[M]^+$ Elemental anal. calcd. for $C_{10}HBr_2FN_2O_3$: C, 31.95; H, 0.27; Br, 42.51; F, 5.05; N, 7.45; O, 12.77. Found: C, 31.95; H, 0.67; N, 7.29; F, 4.90.

Synthesis of 5,8-dibromo-6,7-difluorofuro[3,4-*b*]quinoxaline-1,3-dione (7c)

Compound **7c** was synthesized from compound **6c** (0.50 g, 1.21 mmol) using the same procedure for **7b** and washed with hexane to provide 0.46 g (ca. 96%) as a yellow solid. ^{13}C NMR (75 MHz, acetone, δ, ppm, 25 °C): 158.80, 155.58, 155.31, 152.11, 151.84, 145.61, 141.53, 111.82, 111.66, 111.53. MS(EI) *m*/*z*: 394 $[M]^+$ Elemental anal. calcd. for $C_{10}Br_2F_2N_2O_3$: C, 30.49; Br, 40.57; F, 9.65; N, 7.11; O, 12.18. Found: C, 30.44; N, 6.82.

Synthesis of 5,8-dibromo-6,7-dichlorofuro[3,4-*b*]quinoxaline-1,3-dione (7d)

Compound **7d** was synthesized from compound **6d** (0.28 g, 0.63 mmol) using the same procedure for **7b** and washed with hexane to provide 0.26 g (ca. 97%) as a yellow solid. ^{13}C NMR (75 MHz, acetone, δ, ppm, 25 °C): 158.72, 146.17, 142.79, 140.57, 127.29. MS(EI) *m*/*z*: 427 $[M]^+$ Elemental anal. calcd. for $C_{10}Br_2Cl_2N_2O_3$: C, 28.14; Br, 37.44; Cl, 16.61; N, 6.56; O, 11.25. Found: C, 27.92; N, 6.41; O, 11.62.

Synthesis of 5,8-dibromo-2-hexyl-1H-pyrrolo[3,4-*b*]quinoxaline-1,3(2H)-dione (1: QI-2H-Hex)

Compound **7a** (0.41 g, 1.15 mmol), anhydrous acetonitrile (10 mL), and *n*-hexylamine (0.12 g, 1.15 mmol) were stirred for overnight under an argon atmosphere. After the reaction, the solvent was removed under reduced pressure. After anhydrous DCM (10 mL), anhydrous DMF (1 drop), and oxalyl chloride (1.46 g, 11.5 mmol) were added, the resulting mixture solution was stirred for overnight under an argon atmosphere. The mixture was poured into 100 mL distilled water and extracted with chloroform three times. The organic layer was washed with distilled water and dried with $MgSO_4$. After the removal of the solvent under reduced pressure, the residue was purified by silica gel column chromatography, eluting with DCM to provide 0.39 g (75%) as a yellow solid. ^{1}H NMR (300 MHz, $CDCl_3$, δ, ppm, 25 °C): 8.15 (s, 2H), 3.95 (t, J = 7.2 Hz, 2H), 1.80-1.76 (m, 2H), 1.34-1.32 (m, 6H), 0.88 (t, J = 6.6 Hz, 3 H). ^{13}C NMR (75 MHz, $CDCl_3$, δ, ppm, 25 °C): 162.88, 146.37, 143.18, 136.75, 126.05, 39.47, 31.42, 28.46, 26.66, 22.60, 14.09. MS(EI) *m/z*: 441 $[M]^+$ Elemental anal. calcd. for $C_{16}H_{15}Br_2N_3O_2$: C 43.57, H 3.43, Br 36.23, N 9.53, O 7.25; found: C 43.39, H 3.44, Br 36.30, N 9.48, O 7.44.

Synthesis of 5,8-dibromo-6-fluoro-2-hexyl-1H-pyrrolo[3,4-*b*]quinoxaline-1,3(2H)-dione (2: QI-1F-Hex)

Compound **2** was synthesized from compound **7b** (0.34 g, 0.90 mmol) using the same procedure for **1** and purified by silica gel column chromatography, eluting with DCM to provide 0.36 g (88%) as a yellow solid.

^{1}H NMR (300 MHz, $CDCl_3$, *δ*, ppm, 25 °C): 8.17 (d, *J* = 7.8 Hz, 1H), 3.94 (t, *J* = 7.2 Hz, 2H), 1.80-1.76 (m, 2H), 1.34-1.32 (m, 6H), 0.89 (t, *J* = 3.6 Hz, 3 H). ^{13}C NMR (75 MHz, $CDCl_3$, *δ*, ppm, 25 °C): 163.18, 162.81, 162.72, 159.74, 146.90, 145.61, 143.99, 140.46, 127.08, 126.75, 111.08, 110.80, 39.51, 31.41, 28.46, 26.65, 22.60, 14.09. MS(EI) *m*/*z*: 459 $[M]^+$ Elemental anal. calcd. for $C_{16}H_{14}Br_2F_1N_3O_2$: C 41.86, H 3.07, F 4.14, N 9.15; found: C 41.56, H 3.15, F 4.42, N 9.15.

Synthesis of 5,8-dibromo-6,7-difluoro-2-hexyl-1H-pyrrolo[3,4-*b*]quinoxaline-1,3(2H)-dione (3: QI-2F-Hex)

Compound **3** was synthesized from compound **7c** (0.46 g, 1.17 mmol) using the same procedure for **1** and purified by silica gel column chromatography, eluting with chloroform to provide 0.41 g (73%) as a yellow solid. ^{1}H NMR (300 MHz, $CDCl_3$, *δ*, ppm, 25 °C): 3.95 (t, *J* = 7.5 Hz, 2H), 1.83-1.74 (m, 2H), 1.36-1.26 (m, 6H), 0.89 (t, *J* = 6.9 Hz, 3H). The ^{13}C NMR failed to obtain spectra because of the limited solubility of the product in any solvent such as $CHCl_3$, DMSO, and acetone. MS(EI) *m*/*z*: 477 $[M]^+$ Elemental anal. calcd. for $C_{16}H_{13}Br_2F_2N_3O_2$: C 40.28, H 2.75, Br 33.50, F 7.96, N 8.81; found: C 40.10, H 2.85, Br 33.22, F 8.24, N 8.80.

Synthesis of 5,8-dibromo-6,7-dichloro-2-hexyl-1H-pyrrolo[3,4-*b*]quinoxaline-1,3(2H)-dione (4: QI-2Cl-Hex)

Compound **4** was synthesized from compound **7d** (0.41 g, 0.96 mmol) using the same procedure for **1** and

purified by silica gel column chromatography, eluting with chloroform to provide 0.36 g (73%) as a yellow solid. ^{1}H NMR (300 MHz, $CDCl_3$, δ, ppm, 25 °C): 3.95 (t, J = 7.2 Hz, 2H), 1.83-1.74 (m, 2H), 1.35-1.31 (m, 6H), 0.89 (t, J = 6.9 Hz, 3H). The ^{13}C NMR failed to obtain spectra because of the limited solubility of the product in any solvent such as $CHCl_3$, DMSO, and acetone. MS(EI) m/z: 510 $[M]^+$ Elemental anal. calcd. for $C_{16}H_{13}Br_2Cl_2N_3O_2$: C 37.68, H 2.57, Cl 13.90, N 8.24, O 6.27; found: C 37.46, H 2.78, Cl 13.66, N 8.22, O 6.57.

III. Thermal Properties

The thermal gravimetric analyses (TGA) were performed using a RIGAKU Thermo plus EVO TG8120 thermal analyzer at a heating rate of 10 °C min^{-1} under a N_2 atmosphere. The differential scanning calorimetric (DSC) measurements were performed with an SII EXSTAR DSC-6100 instrument at a heating rate of 5 °C min^{-1} under a N_2 atmosphere. In the DSC measurements, the first cooling and second heating runs were analyzed under a 1 wt% weight loss temperature. The reference sample was Al_2O_3 powder.

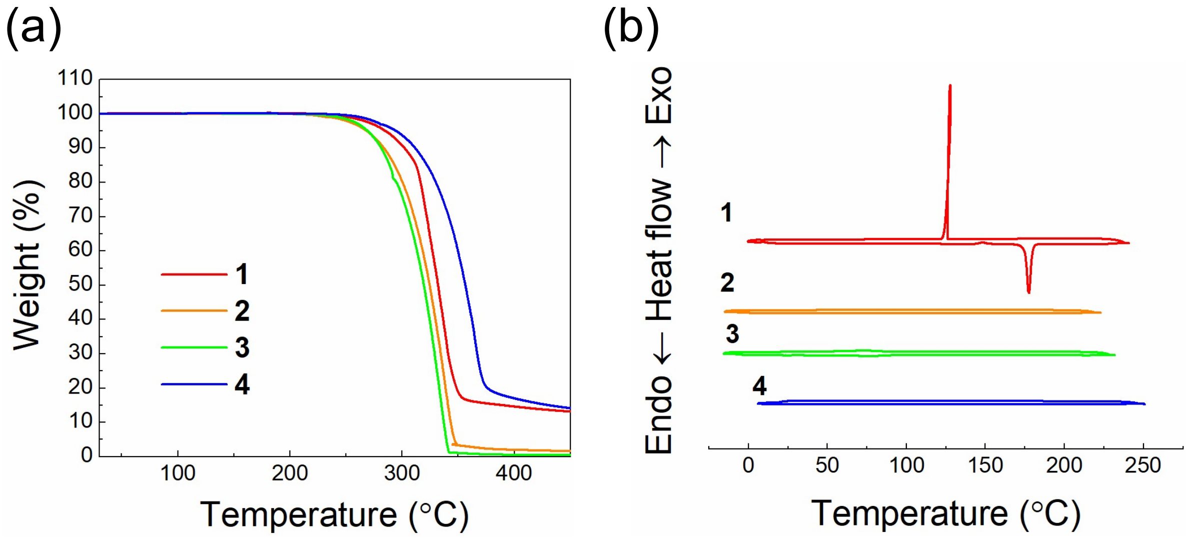

Figure S2. (a) TGA curves of QI-based molecules at the heating rate of 10 ˚C min^{-1} under nitrogen atmosphere. (b) DSC curves of QI-based molecules at the scan rate of 5 ˚C min^{-1} under nitrogen atmosphere. The first cooling and second heating runs were analyzed under 1% weight loss temperature.

IV. Theoretical Calculations

Density Functional Theory (DFT) calculations were performed using the Gaussian 09 program[8] based on the ωB97X-D/6-311G+(d,p)[7] for calculation of the optimized molecular geometry, ground-state electronic structures, and TD-DFT calculations.

Table S1 Absorption data with oscillator strengths over 0.01 estimated by TD-DFT methods for molecules **1-4** at *ω*B97X-D/6-311G+(d,p) optimized geometries.

	**$\lambda_{transition}$ [nm]**	**Oscillator strength**	**Orbital transition contributions**
**1**	338	0.083	HOMO→LUMO (π-π*) (70%)
**2**	334	0.085	HOMO→LUMO (π-π*) (69%)
**3**	326	0.080	HOMO→LUMO (π-π*) (69%)
**4**	336	0.068	HOMO→LUMO (π-π*) (70%)

V. Optical Properties

The UV-vis absorption spectra of the dilute solution and spin-coated thin film of the present compounds were collected using a JASCO Corporation V-670 UV-vis-NIR spectrophotometer.

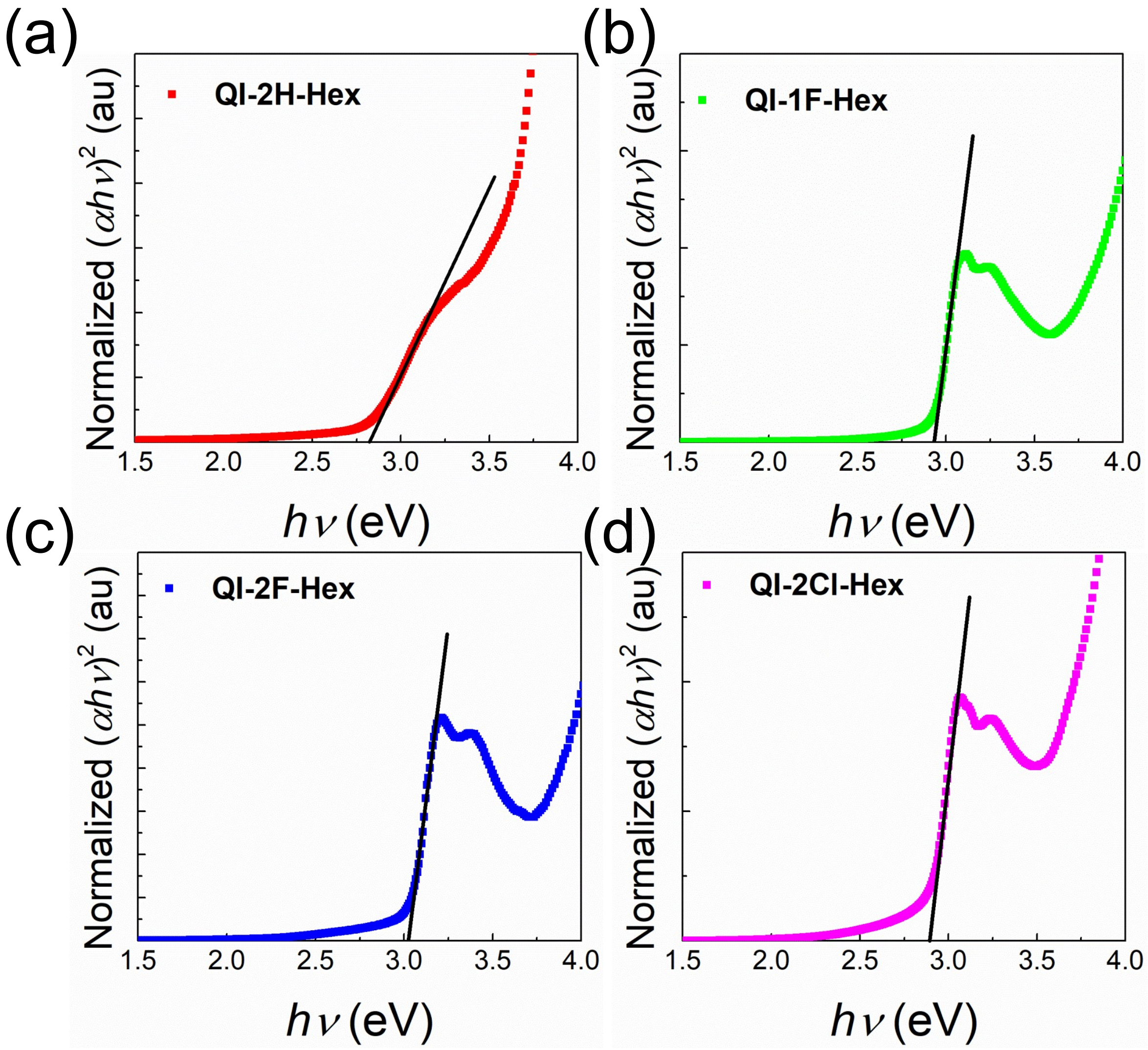

Figure S3 Taut plot from UV-vis absorption spectrum with a linear fit in low photon energy region of (a) **1**, (b) **2**, (c) **3**, and (d) **4**, respectively.

VI. X-ray single-crystal structure analyses

The crystal structures were determined from single-crystal X-ray diffraction data. The measurements were made by a RIGAKU R-AXIS RAPID II imaging plate with Cu-Kα radiation from a rotation anode source with a confocal multilayer X-ray mirror (RIGAKU VM-spider, λ = 1.54187 Å). The diffraction data were corrected under −100 °C. The structures were solved by the direct method (SHELX-97). The structures were refined by the full-matrix least-squares procedure by applying anisotropic temperature factors. The single crystals were grown via the slow diffusion of poor solvents into good solvent solutions of QI molecules: hexane into dichloromethane solution for **1**, hexane into chloroform solutions for **2-4**. The positions of the hydrogen atoms were calculated.

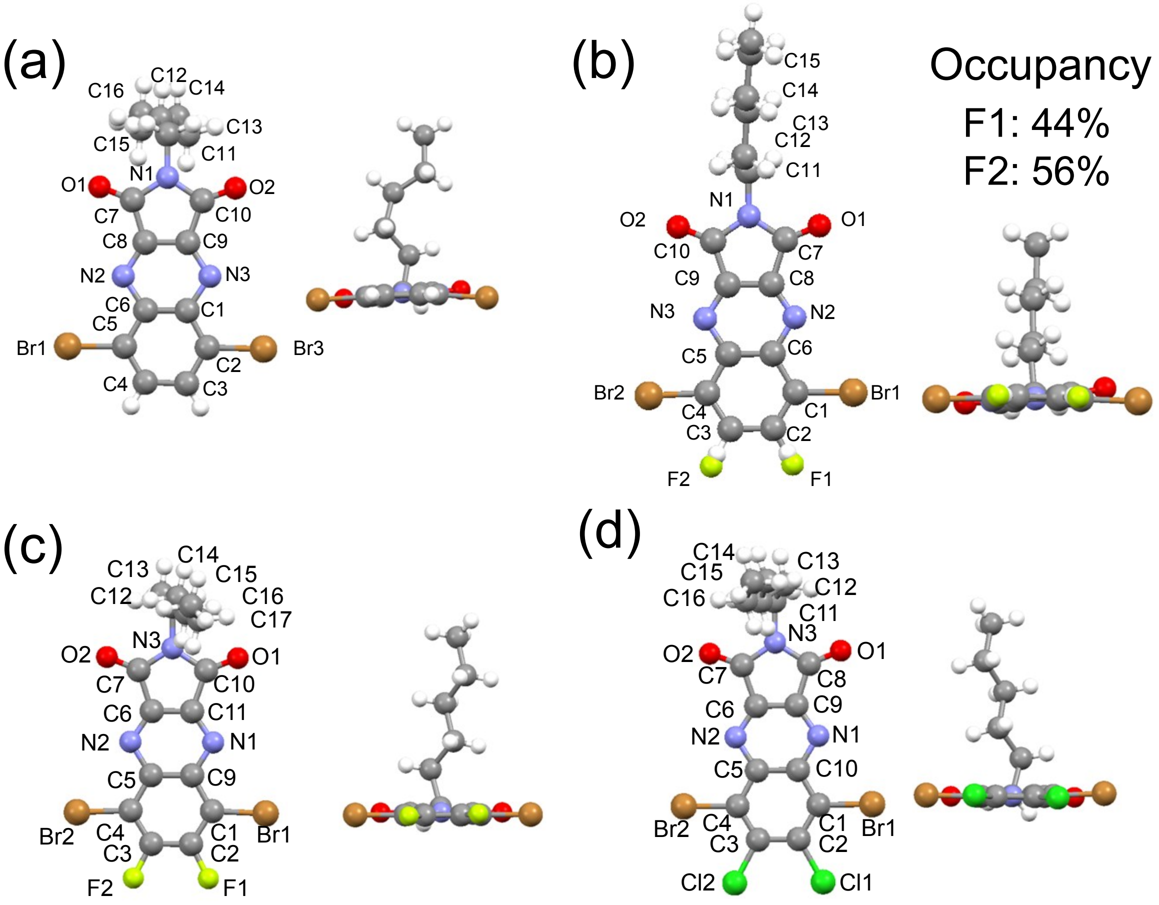

Figure S4 Molecular structures and geometries of (a) **1**, (b) **2**, (c) **3**, and (d) **4**, respectively.

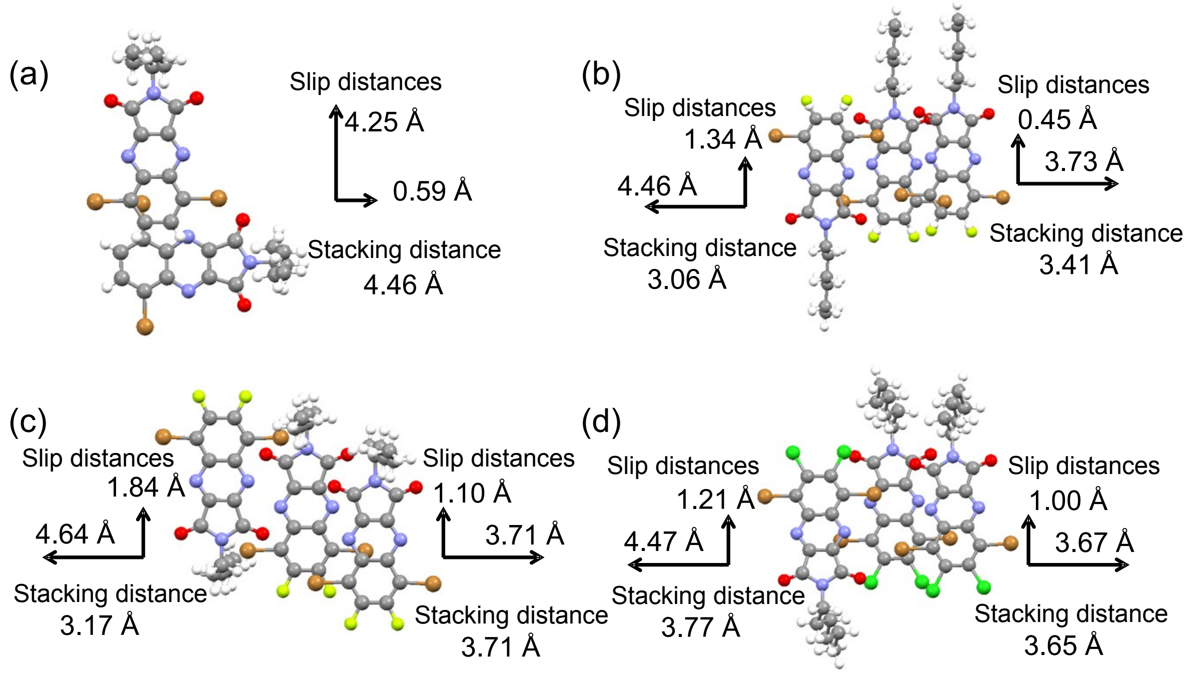

Figure S5 Overlap structures of (a) **1**, (b) **2**, (c) **3**, and (d) **4**, respectively.

Table S2 Crystallographic data of molecules **1-4**.

	**1**	**2**	**3**	**4**
Chemical formula	$C_{16}H_{15}Br_2N_3O_2$	$C_{16}H_{14}Br_2F_1N_3O_2$	$C_{16}H_{13}Br_2F_2N_3O_2$	$C_{16}H_{13}Br_2Cl_2N_3O_2$
Crystal system	Monoclinic	Monoclinic	Monoclinic	Monoclinic
Formula weight	441.12	459.12	477.10	510.01
Sharp	Pale yellow plate	Pale yellow plate	Pale yellow plate	Pale yellow plate
Space group	*Pc*	$P2_1/c$	$P2_1/c$	$P2_1/c$
a (Å)	5.7237(2)	14.9939(3)	13.5124(3)	14.8869(3)
b (Å)	16.2252(7)	16.3893(3)	16.6954(3)	16.2742(3)
c (Å)	8.9938(4)	6.87863(12)	7.74975(14)	7.56800(14)
α (°)	90.0000	90.0000	90.0000	90.0000
β (°)	97.574(2)	97.4348(11)	106.5690(10)	105.4340(8)
γ (°)	90.0000	90.0000	90.0000	90.0000
V (Å^3)	827.95(6)	1676.14(5)	1675.71(6)	1767.40(6)
Z	2	4	4	4
D_{cal} (g cm^{-3})	1.769	1.819	1.891	1.917
Data/parameters	2729/208	3019/227	3054/226	3238/226
Goodness of fit on F_0^2	1.098	1.008	1.024	1.060
R_1^a/R_W^b	0.0766/0.1893	0.0654/0.1257	0.535/0.1246	0.0469/0.1049
Reflection used	2729	3019	3054	3238

^{a} $R_1 = \Sigma||F_0| - |F_c|| / \Sigma|F_0|$. ^{b} $R_W = [\Sigma\omega(|F_0| - |F_c|)^2 / \Sigma\omega F_0^2]^{1/2}$.

VII. Fabrication and Characterization of Organic Field-Effect Transistors

A 300-nm thick thermally grown SiO_2 layer on a highly doped silicon wafer (capacitance (C_i) = 11.5 nF cm^{-2}) was cleaned in a piranha solution (70% H_2SO_4 + 30% H_2O_2) for 15 min and then washed with distilled water.[9] As a gate insulator layer, the TTC (capacitance = 106 nF cm^{-2})[10] was thermally evaporated to form a 20 nm thick TTC layer on the Si substrate. The resulting gate insulator capacitance was 10.4 nF cm^{-2}. The active layer was formed by thermal deposition under a vacuum of ca. 1.0×10^{-4} Pa, in which the resulting thickness was 45 nm. Finally, the source and drain electrodes were formed by the vapor deposition of Au (50 nm). The standard channel length (L) and width (W) were 50 μm and 1000 μm, respectively. The organic field-effect transistor (OFET) properties were measured by a Keithley 4200 semiconductor parameter analyzer under vacuum conditions. The field-effect mobility (μ) and threshold voltage (V_{th}) were calculated in the saturation region using the equation $I_{DS} = \mu(WC_i/2L)(V_G - V_{th})^2$, where I_{DS} and V_G are the drain current and gate voltage, respectively. The capacitance including the TTC layer on the SiO_2 substrate was calculated to be 1.25×10^{-8} F cm^{-2}.

The grazing-incidence wide-angle X-ray scattering (GIWAXS) measurements were carried out at BL40B2 in SPring-8 (Hyogo, Japan). The wavelength of the X-ray beam was 0.1 nm, and the camera length was 330 mm. Two-dimensional scattering images were acquired using a photon-counting detector (Pilatus3 2M, Dectris, Ltd.). The samples were mounted in a helium cell to reduce radiation damage. The

data acquisition time is 10 s. GIWAXS was measured at an incident angle of 0.10 degrees, which was lower than the critical angle of total external reflection at the silicon surface. Therefore, the incident X-rays pass through the sample and reflect on the silicon wafer surface. The components of the scattering vector, q, parallel and perpendicular to the sample surface were defined as $q_y = (2\pi/\lambda)\sin(2\theta)\cos(\alpha_f)$ and $q_z = (2\pi/\lambda)(\sin(\alpha_i) + (\sin(\alpha_f))$, respectively. Here, α_i is the incident angle of the X-ray beams, α_f is the exit angle with respect to the surface, λ is the X-ray wavelength and 2θ is the angle between the scattered beam and the plane of incidence.[11]

The atomic force microscopy (AFM) images were obtained using an SII Nanonavi SPA400 scanning probe microscope with an SII SI-DF40 cantilever.

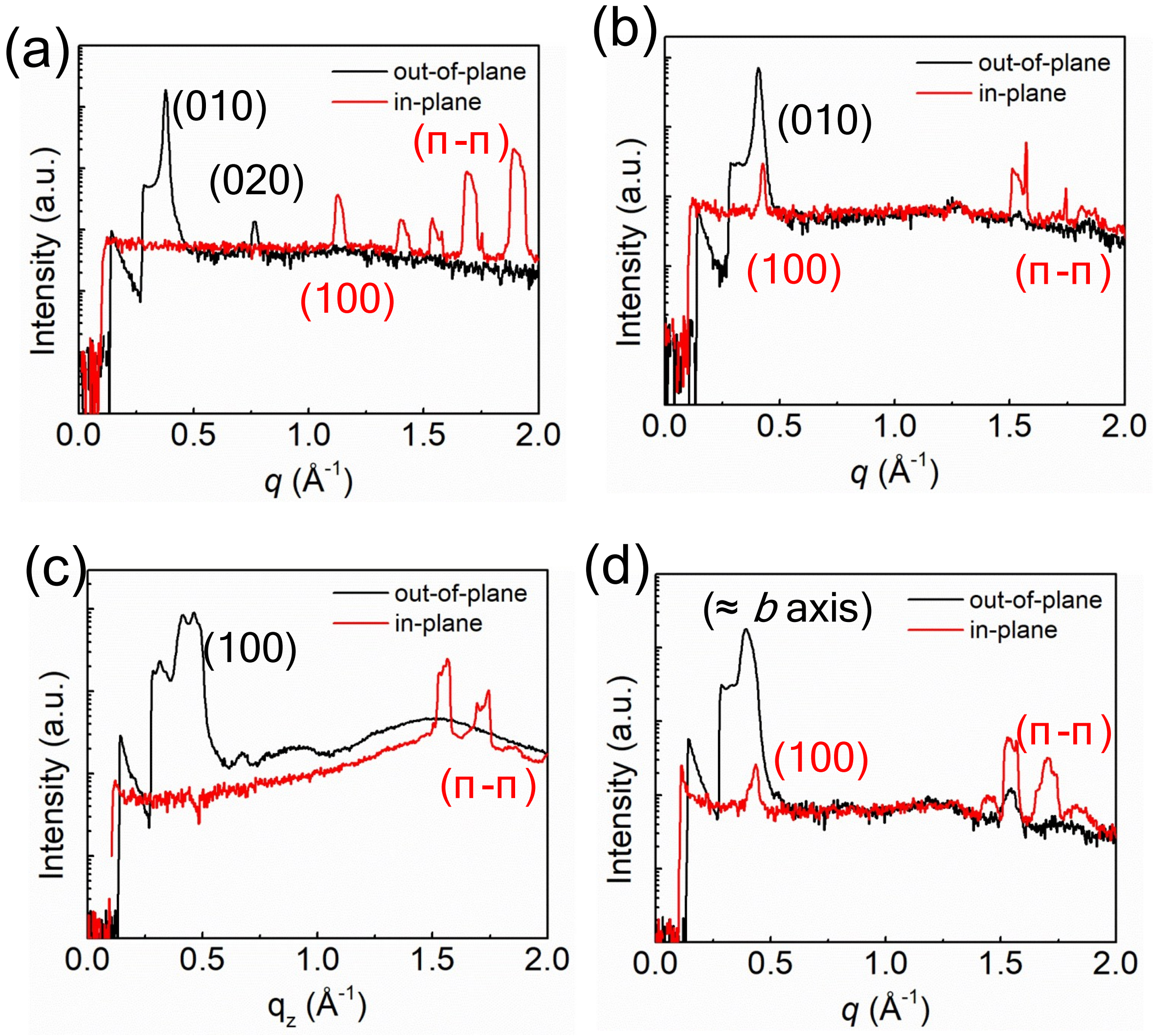

Figure S6 GIWAXS one-direction profiles of (a) **1**, (b) **2**, (c) **3**, and (d) **4**, respectively.

Table S3 Packing parameters of **1-4** thin films extracted from GIWAXS patterns.

		**Out-of-plane**			**In-plane**	
		q_z [Å^{-1}]	*d*-spacing [Å]		q_{xy} [Å^{-1}]	*d*-spacing [Å]
**1**	(010)	0.38	16.5	(100)	1.12	5.60
				(π-π)	1.69	3.73
**2**	(010)	0.41	15.3	(100)	0.43	14.8
				(π-π)	1.74	3.60
**3**	(100)	0.46	13.6	(π-π)	1.74	3.60
**4**	(010)	0.39	16.1	(100)	0.43	14.6
				(π-π)	1.70	3.69

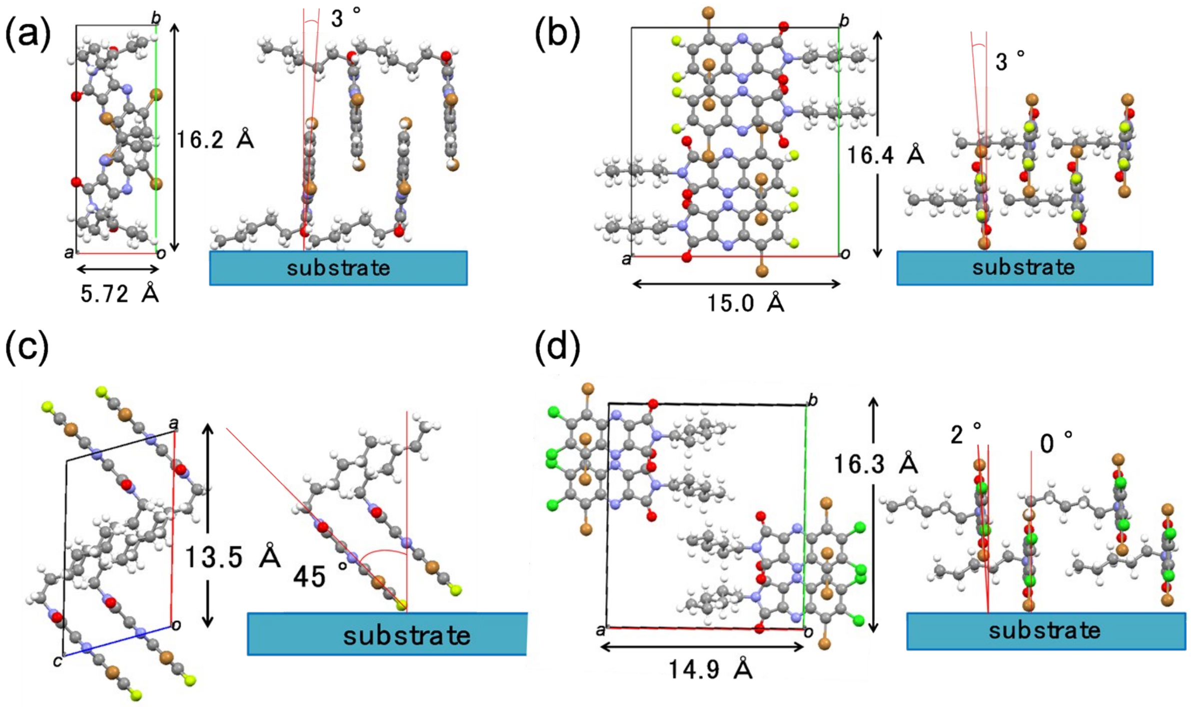

Figure S7. Molecular arrangements on the substrate of (a) **1**, (b) **2**, (c) **3**, and (d) **4**.

Table S4 OFET performances for molecules **1-4**.

	n-channel		
	$\mu_{e,avg}$ ($\mu_{e,max}$) [$cm^2V^{-1}s^{-1}$]	V_{th} [V]	I_{on}/I_{off}
**1**	1.4×10^{-3} (1.8×10^{-3})	77.4	3.0×10^{3}
**2**	1.8×10^{-4} (2.9×10^{-4})	69.4	2.8×10^{3}
**3**	4.3×10^{-3} (6.9×10^{-3})	69.4	1.8×10^{4}
**4**	7.1×10^{-3} (7.7×10^{-3})	67.6	1.7×10^{3}

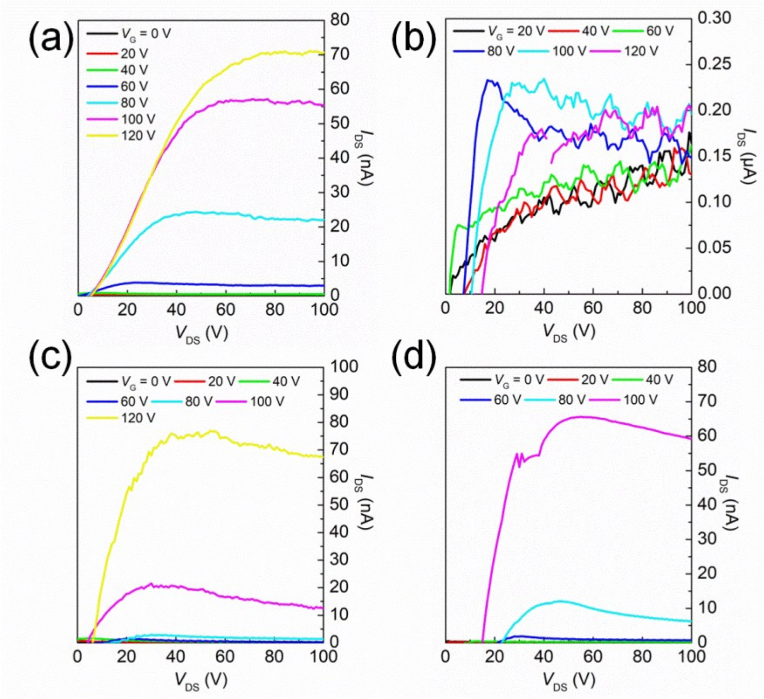

Figure S8 n-Channel output curve of (a) **1**, (b) **2**, (c) **3**, and (d) **4**, respectively.

References

1. J. Kim, S. H. Park, J. Kim, S. Cho, Y. Jin, J. Y. Shim, H. Shin, S. Kwon, I. Kim, K. Lee, A. J. Heeger, and H. Suh, *J. Polym. Sci. Part A*, 2011, **49**, 369-380.

2. G. K. Dutta, T. Kim, H. Choi, J. Lee, D. S. Kim, J. Y. Kim, and C. Yang, *Polym. Chem*., 2014, **5**, 2540-2547.

3. Z. Chen, P. Cai, J. Chen, X. Liu, L. Zhang, L. Lan, J. Peng, Y. Ma, and Y. Cao, *Adv. Mater*., 2014, **26**, 2586-2591.

4. X. Gao, J. Shen, B. Hu, and G. Tu, Macromol. *Chem. Phys*., 2014, **215**, 1388-1395.

5. P. Gawrys, T. Marszalek, E. Bartnik, M. Kucinska, J. Ulanski, and M. Zagorska, *Org. Lett*., 2011, **13(22)**, 6090-6093.

6. T. Hasegawa, K. Aoyagi, M. Ashizawa, Y. Konosu, S. Kawauchi, N. S. Sariciftci, and H. Matsumoto, *Chem. Lett*., 2015, **44**, 1128-1130.

7. M. J. Frisch, G. W. Trucks, H. B. Schlegel, G. E. Scuseria, M. A. Robb, J. R. Cheeseman, G. Scalmani, V. Barone, B. Mennucci, G. A. Petersson, H. Nakatsuji, M. Caricato, X. Li, H. P. Hratchian, A. F. Izmaylov, J. Bloino, G. Zheng, J. L. Sonnenberg, M. Hada, M. Ehara, K. Toyota, R. Fukuda, J. Hasegawa, M. Ishida, T. Nakajima, Y. Honda, O. Kitao, H. Nakai, T. Vreven, J. A. Montgomery Jr, J. E. Peralta, F. Ogliaro, M. Bearpark, J. J. Heyd, E. Brothers, K. N. Kudin, V. N. Staroverov, R. Kobayashi, J. Normand, K. Raghavachari, A. Rendell, J. C. Burant, S. S. Iyengar, J. Tomasi, M. Cossi, N. Rega, N. J. Millam, M. Klene, J. E. Knox, J. B. Cross, V. Bakken, C. Adamo, J.

Jaramillo, R. Gomperts, R. E. Stratmann, O. Yazyev, A. J. Austin, R. Cammi, C. Pomelli, J. W. Ochterski, R. L. Martin, K. Morokuma, V. G. Zakrzewski, G. A. Voth, P. Salvador, J. J. Dannenberg, S. Dapprich, A. D. Daniels, Ö. Farkas, J. B. Foresman, J. V. Ortiz, J. Cioslowski and D. J. Fox, The molecular orbital calculation was carried out using the Gaussian 09, Revision B.01, Gaussian, Inc., Wallingford CT, 2009.

8. J.-D. Chai, and M. Head-Gordon, *Phys. Chem. Chem. Phys*. 2008, **10**, 6615–6620.

9. J. K. Seu, A. P. Pandey, F. Haque, E. A. Proctor, A. E. Ribbe, J. S. Hovis, *Biophys. J.*, 2007, **92 (7)**, 2445-2450.

10. M. Kraus, S. Richler, A. Opitz, W. Brütting, S. Haas, T. Hasegawa, A. Hinderhofer, F. Schreiber, *J. Appl. Phys*., 2010, **107**, 094503.

11. T. Fujisawa, K. Inoue, T. Oka, H. Iwamoto, T. Uruga, T. Kumasaka, Y. Inoko, N. Yagi, M. Yamamoto, T. Ueki, *J. Appl. Crystallogr.*, 2000, **33**, 797-800.
